# Who Seeks Clear Aligner Therapy? A European Cross-National Real-World Data Analysis

**DOI:** 10.3390/life13010065

**Published:** 2022-12-25

**Authors:** Martin Baxmann, Lan Huong Timm, Falk Schwendicke

**Affiliations:** 1 Orthodentix, Arnoldstrasse 13b, 47906 Kempen, Germany; 2 DrSmile-DZK Deutsche Zahnklinik GmbH, Königsallee 92a, 40212 Düsseldorf, Germany; 3 Department of Oral Diagnostics, Digital Health and Health Services Research, Charité—Universitätsmedizin Berlin, Aßmannshauser Straße 4-6, 14197 Berlin, Germany

**Keywords:** orthodontics, orthodontic treatment, adult treatment, clear aligners, malocclusion, motivating factors, dental practice management, orthodontic diagnosis, cross-country comparisons

## Abstract

A key step prior to clear aligner therapy (CAT) is the clinical examination and case selection, which includes understanding the specific orthodontic problem to be managed and the wider evaluation of oral health. Seeking CAT may further differ along sociodemographic parameters or across countries, as may perceived orthodontic treatment needs and oral health. We aimed to characterize patients seeking CAT across five European countries. Anonymized real-life data from one large CAT provider (DrSmile, Berlin, Germany) was retrospectively sampled for the period 1 November 2021–31 December 2021. A total of 15,015 patients (68.4% females, 31.6% males, with an age range of 18–81 years, median 30.0 years) were included. The cross-national comparison revealed a significant difference in gender distribution (*p* < 0.001/Chi-square), with the highest proportion of males in Italy (434/1199, 36.2%) and the lowest in Poland (457/1600, 28.6%); generally, more females sought CAT. The largest motivational factor in all countries for seeking CAT was crowding, in both males and females. By and large, patients paid out of pocket for CAT. The prevalence of caries, periodontitis, and craniomandibular dysfunction as well as the numbers of missing teeth were generally low, albeit with significant differences between sociodemographic groups and countries for caries and periodontitis. Patients seeking CAT showed a low prevalence in oral conditions but differed in their sociodemographic characteristics across countries. Dentists and orthodontists should consider these country-specific differences when planning CAT.

## 1. Introduction

Orthodontics is a branch of dentistry specializing in the diagnosis, prevention, and treatment of irregularities of the teeth and malocclusion in both children and adults [1]. It has been shown that malocclusion can increase the prevalence of tooth decay and affect periodontal health [2,3,4].

The prevalence of dental malocclusion and orthodontic treatment need has been assessed in several epidemiological studies, mainly in children and adolescents [5,6,7,8], but less frequently in adults. These studies usually use survey designs to estimate the need for orthodontic therapy [9,10,11] but suffer from being relatively small and only representative for specific populations or countries (at best).

Clear aligner therapy (CAT) is based on clear, thermoformed plastic trays for the correction of from mild to moderate tooth misalignments and is becoming increasingly popular in adult orthodontics for the correction of from mild to moderate forms of malocclusion [12,13,14], as it is more comfortable and less obstructive to oral hygiene and other functions compared with fixed orthodontic appliances and can achieve significant improvement when diagnosed and planned carefully [15,16]. A growing awareness of oral health, the wish to increase dental aesthetics, and psychosocial factors have been found to affect the demand for adult orthodontics (not only CAT) [17,18,19], which is by and large paid for out-of-pocket and not covered by public health services.

A central step prior to active CAT is a clinical examination and case selection, including the evaluation of the specific orthodontic problem to be solved but also the presence of caries, periodontitis, or craniomandibular dysfunction (CMD), all of which would need to be addressed beforehand. Currently, it is not clear what the clinical status of patients seeking CAT is, and there is also limited knowledge of if in different countries, patients seeking CAT differ (not only regarding the clinical but also the described sociodemographic parameters and needs). A cross-national comparison might reveal that patients seeking CAT present different characteristics, perceived orthodontic treatment needs, and oral health statuses, all of which affect treatability with CAT.

The present study aimed to assess the sociodemographic characteristics, orthodontic treatment needs, and dental status needs of adults in Austria, Germany, Spain, Italy, and Poland. We further compared these aspects in different age and gender groups across the countries.

This could be relevant information for healthcare decision-makers to prioritize interventions and decisions about orthodontic appliances, considering the increasing demand for orthodontic treatment.

## 2. Materials and Methods

### 2.1. Study Design

This retrospective study was conducted using anonymized data provided by DrSmile, a brand of Urban Technology GmbH (Berlin, Germany). DrSmile is a Berlin-headquartered health tech company providing CAT, including interproximal enamel reduction (IPR) and attachments. It operates a dental platform with a network of more than 550 local partner dentists and orthodontists across Europe. The platform provides dentists with a centralized location to store patient records and information. The dental operating system secures medical and dental records and prescriptions and includes features that help dentists to serve their patients better. All DrSmile dentists and orthodontists are licensed to practice and are trained upfront as well as continuously (“DrSmile Academy”). The training is designed to ensure that all dentists are sufficiently trained to perform from simple to moderately difficult CAT and to conduct a standardized clinical examination for appropriate case selection within the DrSmile treatment scope.

Using pseudonymized, routinely collected data from electronic dental records and patient medical forms, we gathered data on patients’ sociodemographic parameters, their main motivational factors for seeking CAT, as well as the prevalence of missing teeth, caries, craniomandibular dysfunction (CMD), and/or periodontal disease, and other contraindications to CAT.

Our study was conducted in accordance with the World Medical Association Declaration of Helsinki and reporting was performed according to the STROBE guideline [20]. The data were collected as part of routine patient care and anonymized for health and care research, which does not require the approval of an ethics committee or informed consent according to the Berlin State Hospital Act (Landeskrankenhausgesetz Berlin) and the recommendations of the Datenschutz und IT-Sicherheit im Gesundheitswesen (DIG) task force of the German Association for Medical Informatics, Biometry, and Epidemiology (GMDS). The orthodontic diagnostic procedures followed the “Clinical Practice Guidelines for Orthodontics and Dentofacial Orthopedics” of the American Association of Orthodontists (AAO).

### 2.2. Participants

A comprehensive sample of patients who attended an initial examination and sought CAT between 1 November 2021 and 31 December 2021 was drawn, and patients were included regardless of whether they were eligible for DrSmile CAT or deemed ineligible for treatment after the examination.

### 2.3. Data Collection and Variables

A clinical examination of the hard and soft tissues was performed within the dental chair using routine practice equipment. All teeth were inspected, and a record of the findings was saved. Basic periodontal examinations (BPE) [21,22], CMD screenings [23], as well as radiographs according to the recommendations of the British Orthodontic Society were performed [24] to rule out contraindications to CAT, such as CMD or active periodontal disease. The orthodontic examinations followed the “Clinical Practice Guidelines for Orthodontics and Dentofacial Orthopedics” of the American Association of Orthodontists (AAO) [25]. Contraindications and reasons for rejection of CAT, if any, were recorded by the dentists. From these variables, data on the prevalence of caries, periodontitis, CMD, and missing teeth were gathered. Missing wisdom teeth were not considered “missing teeth”.

In addition, patients were asked to indicate in the medical forms whether they were taking medication and/or had systemic diseases. Patients were further asked to indicate their main motivating factors for seeking CAT.

### 2.4. Bias

While recruitment of DrSmile patients carries the risk of selection bias, we drew a comprehensive sample from the pool of all attending patients, with the only exclusion criterion being age (individuals aged <18 years were excluded).

### 2.5. Statistical Analysis

We conducted descriptive analyses of the variables, and two-sided chi-squared tests were used for the statistical analysis. The data were analyzed with JASP 0.16.3 (University of Amsterdam, Amsterdam, The Netherlands). The level of significance was determined to be *p* < 0.05.

## 3. Results

### 3.1. General Patient Characteristics

We present data for patients seeking CAT between 1 November 2021 and 31 December 2021 in five countries in Europe (Austria, Germany, Spain, Italy, and Poland). The total sample size was 15,015 patients and available for analysis without exclusion (comprehensive sample). Of these, 10,277/15,015 (68.4%) were female patients and 4738/15,015 (31.6%) were male patients. The median age was 30.0 years (with a range of 18–81). By age group, older adults formed the smallest group (older than 55 years, *n* = 381, 2.5%), followed by middle-aged adults (36–55 years, *n* = 3889, 25.9%), while young adults (18–35 years, *n* = 10,745, 71.6%) formed the largest group.

Of all the patients, the majority (*n* = 6722, 44.7%) were German patients, 29.5% (*n* = 4425) were Spanish, 10.7% (*n* = 1600) were Polish, 8.0% (*n* = 1199) were Italian, and 7.1% (*n* = 1069) were Austrian patients.

**Figure 1:**
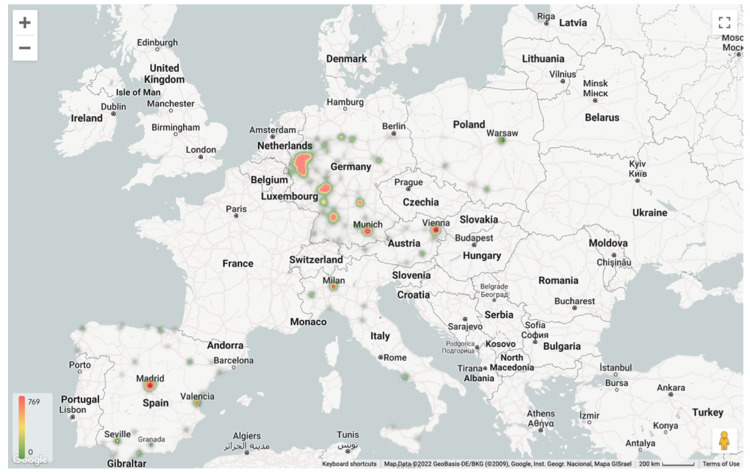
Number of patients seen. The heatmap shows the number of patients in different zip code areas (according to patients’ residences) in the five countries.

The longitudes and latitudes of all the patients’ residences were calculated and entered into a computing-based data cloud (Snowflake Inc., Bozeman, MT, USA) and visualized using a business intelligence software (Looker Data Sciences Inc., Santa Cruz, CA, USA) via Google Maps (Google LLC, Mountain View, CA, USA). The distribution of patients according to their place of residence is shown in Figure 1.

### 3.2. Cross-National Comparison

#### 3.2.1. Sociodemographic Characteristics

The median age in Austria was 31.0 years (with a range of 18–74), in Germany 30.0 years (with a range of 18–79), in Spain 31.0 (with a range of 18–81), in Italy 29.0 (with a range of 18–76), and in Poland 31.0 years (with a range of 18–79). By age group, young patients formed the largest group in all the countries, followed by middle-aged adults, whereas older adults formed the smallest group in all the countries.

The cross-national comparison revealed a significant difference in gender distribution (*p* < 0.001), with the highest proportion of males in Italy (434/1199, 36.2%) and the lowest in Poland (457/1600, 28.6%); overall, females were more frequent in all the countries.

While the 18–35-year-old age group was the largest group in all the countries, patients were more likely to be in the 18–35-year-old age group in Italy (895/1199, 74.7%) and Germany (4969/6722, 73.9%) than in the other countries, where the proportions were less than 70.0% (*p* < 0.001) (Table 1).

#### 3.2.2. Motivations for Seeking CAT

The majority of all the patients (38.1%, 5727/15,015 respondents) considered “crowding” their most important motivation factor for seeking CAT, while 3671/15,015 respondents (24.5%) indicated “other functional or dental health” motives, 2926/15,015 (19.5%) respondents indicated “protruding teeth”, 1853/15,015 (12.3%) respondents indicated “tooth spacing”, and 838/15,015 (5.6%) respondents did not answer the question.

The cross-national comparison revealed significant differences in the main motives for seeking CAT, with patients in Spain (2005/4425, 45.3%) and Italy (505/1199, 42.1%) reporting “crowding” more frequently than patients in Germany (2231/6722, 33.2%) and Austria (386/1069, 36.1%) (*p* < 0.001). Tooth “spacing” was more frequently reported as one of the motives in Germany (1000/6722, 14.9%) than in Poland (133/1600, 8.3%) (Table 2).

Males were more likely to report “spacing” as the main motive, whereas females were more likely to report “crowding” or “protruding teeth” as their main motives for seeking orthodontic treatment (*p* < 0.001) (Appendix A Table A1).

#### 3.2.3. Insurance Type

The majority of all the patients (76.9%, 11,539/15,015) were patients with statutory health insurance, while 2638/15,015 patients (17.6%) were patients with private health insurance, and 838/15015 patients (5.6%) did not answer the question.

In the cross-national comparison, there was a significant difference in the types of insurance, with Spanish patients being significantly more likely to have private health insurance (1331/4425, 30.1%), while Austria (103/1069, 9.6%) and Germany (696/6722, 10.4%) had the smallest proportion of patients with private health insurance (*p* < 0.001) (Appendix A Table A2).

#### 3.2.4. Oral Health

Significant differences between the >55 years age group and the other age groups (18–35 years and 36–55 years) were found in the prevalence of periodontitis, with older patients more likely to have periodontal disease (39/381, 10.2%) than the 36–55 years age group (78/3889, 2.0%) and the 18–35 years age group (57/10,745, 0.5%) (*p* < 0.001).

Furthermore, a significant difference in caries prevalence was found between age groups. Its frequency decreased with age, with younger patients in the 18–35 years age group (163/10745, 1.5%) more likely to have untreated carious lesions than older patients in the 36–55 years age group (35/3889, 0.9%) and the >55 years age group (2/381, 0.5%) (*p* < 0.05).

No significant differences between the patient age groups were found for CMD (*p* = 0.668).

The prevalence of periodontitis was higher in males than in females (*p* < 0.001). There were no significant differences in the prevalence of CMD (*p* = 0.113) or dental caries (*p* = 0.291) between males and females at the time of the initial assessment for CAT.

In the cross-national comparison, there was a significant difference in the prevalence of untreated carious lesions (98/4425, 2.2%) and periodontitis (134/4425, 3.0%) among Spanish patients compared with the other countries (*p* < 0.001). No significant differences were found for CMD between the countries (*p* = 0.306) (Table 3).

Spanish patients (230/4425, 5.2%) were significantly more likely to have missing teeth than patients in the other countries (*p* < 0.05) (Table 4).

Generally, the prevalence of oral conditions was low.

Significant differences between the >55 years, the 35–55 years, and the 18–35 age groups were found, with older patients in the >55 years age group (32/381, 8.4%) and 35–55 years age group (199/3889, 5.12%) significantly more often missing teeth than younger patients (256/10,745, 2.38%) (*p* < 0.001). No significant differences were found between genders (*p* = 0.612) (Appendix A Table A3).

## 4. Discussion

In adult orthodontics, there is an increasing demand for CAT to correct from mild to moderate forms of malocclusion. A key step prior to CAT is an in-depth clinical examination and appropriate case selection. This includes understanding the specific orthodontic problem to be resolved and assessing if findings such as caries, periodontitis, or CMD require pretreatment and hence postponement of CAT until after they are resolved. So far, it remains unclear if the motivations for seeking CAT but also the sociodemographic parameters and oral health of potential CAT patients differ across countries. This study aimed to compare the sociodemographic characteristics, motivations for seeking CAT, and the oral health of 15,015 patients seeking CAT in Austria, Germany, Spain, Italy, and Poland.

In the present sample, adults seeking CAT were mostly younger than 35 years and female; this was consistent across the countries. Notably, the proportion of males and older patients differed to some degree, e.g., males attended significantly more often in Italy than in the other countries. Moreover, spacing and crowding were the main motivational factors for seeking CAT, again with differences between the countries. The oral health of potential CAT patients was generally high, again with differences between sociodemographic groups but also countries.

To our knowledge, this is the first study to examine the cross-national sociodemographics and oral health of adult European patients seeking CAT on a larger scale with real-time medical data. Our findings need in-depth exploration.

First, the results of the present study suggested that there are significant differences between older and younger patient groups in the prevalence of both dental caries and periodontitis. In agreement with most studies [26,27], the prevalence of caries was found to be higher at younger ages and to decrease with age across all countries [28,29], while the opposite was true for periodontitis [30,31]; we also confirmed differences between genders [30,32,33,34]. Generally, oral health was good in comparison with representative samples from national surveys, likely to be because patients seeking CAT are different from the national average, i.e., more aware of their oral health and interested in maintaining or, more likely, improving it. Moreover, they may also differ in their socioeconomic status (especially as CAT is mainly paid out-of-pocket), with access to oral care but also oral health literacy differing from that of other groups.

The low prevalence of oral conditions, particularly periodontitis, is assuring. While orthodontic therapy is increasingly seen as facilitating supportive periodontal care [35,36], most studies still suggest that prior to orthodontic therapy, active periodontitis should be resolved [37,38] and that reduced orthodontic velocity and force application should be considered to reflect the specific biomechanical needs in periodontitis patients [38,39,40].

The cross-national comparison showed a significant difference in the prevalence of untreated carious lesions and periodontitis in Spanish patients compared to the other countries, with Spanish patients much more likely to have active oral disease. In addition, we found that Spanish patients were significantly more likely to have missing teeth than patients in the other countries (although, the number of patients missing more than five teeth was highest in Poland). These differences in oral health and status may reflect not only the healthcare organization in each country (Austria and Germany had the largest proportion of patients with statutory insurance, while Spain had the largest proportion of patients with private insurance) but also the related amount of capita spending for healthcare, which was lower in Poland and Spain than Austria and Germany. From a research perspective, it is interesting to see that our findings align with the data from other sources [41,42,43], confirming that Spanish adults show a higher proportion of unmet dental treatment needs (5.4%) compared with the EU and Eurozone (4.1% and 3.9%, respectively) [44,45]. Data from routine sources such as ours may, hence, be used to triangulate, but also to monitor, oral health in short intervals and large samples, something which national surveys cannot.

Second, a significant difference in gender distribution was found between the countries, while generally, more females sought CAT in all the countries. We found the highest proportion of males in Italy and the lowest in Poland. These cross-national differences in self-perception and self-care are in line with a large global survey conducted among 27,000 internet users aged over 15 in 22 countries. The survey showed significant gender differences in all countries, with females spending more hours on personal care than males. In the EU, Italian men in particular spend the most hours on personal care, while Polish men spend the least [46,47].

Third, crowding was reported by most patients as the main motive for seeking CAT, with differences between the countries, i.e., Spanish and Italian patients reported this motive more frequently than German and Austrian patients, while spacing was reported more often by German and Austrian patients. Differences in motives between genders were found, with males more likely to report spacing as the primary motive, whereas females more frequently indicated crowding as the main motivational factor for seeking CAT. The differences between populations are consistent with a review by Cenzato et al., who analyzed 14 studies and pointed to differences between different populations caused by both genetic factors and environmental influences in the development of malocclusion traits. Furthermore, in agreement with our results, they found crowding to be one of the most common malocclusion traits across all the populations and genders, followed by spacing [48].

This study comes with several limitations. First, the validity of this study is limited by the retrospective study design. This study includes a large sample of patients over the age of 18 but does not include children and adolescents and includes only a small number of older patients over the age of 56. Moreover, it stemmed from a specific patient pool seeking CAT at one provider in five countries, and it may not be representative of patients seeking other orthodontic therapy at other providers, and with all likelihood, it will not allow inferring for other countries (as we confirmed cross-country differences).

Second, we included larger samples of German and Spanish patients, while the numbers of patients from the other countries varied. This was due to different population sizes, but also the fact that partner practices were not available in similar densities across all the countries. To reduce the risk of selection bias in the selection of patients for this study, we opted for the same inclusion criteria for different nationalities and for the inclusion of the entire patient group without sampling.

Last, all the examiners underwent extensive training on the same platform (“DrSmile Academy”) to ensure a standardized clinical examination for an appropriate case selection in CAT. However, cross-national differences in dental education in the EU may result in different examination schemes, although a mutual recognition of qualifications in the EU shows that dental education is largely similar. While the goal was to ensure that each examiner assessed consistently with his or her peers, a high degree of consistency between examiners cannot guarantee interrater reliability due to the lack of a calibration process.

Further studies are needed to evaluate other factors such as the type of malocclusion and to include greater heterogeneity among patients to improve statistical power.

## 5. Conclusions

Adult patients who are dissatisfied with their appearance are more likely to seek clear aligner therapy. Genders and age groups likely had an impact on the patients’ desire for CAT. Patients who were seeking CAT treatment appeared to have better periodontal status than the general population, and caries prevalence appeared to be lower in these patients. There were significant cross-national differences in the prevalence of caries and periodontitis in Europe, and age had an impact on the prevalence of dental health problems present at the time of the initial consultation across all the countries.

The patient’s motivation to seek orthodontic treatment should be considered during treatment planning to increase the chances of a mutually satisfactory treatment outcome. It is important to consider the patient’s dental health during treatment planning.

This research can help dentists, orthodontists, and orthodontic societies use information about patients’ current needs and requirements to prepare and plan for orthodontic treatment.

## Figures and Tables

**Table 1:** Distributions of sociodemographic variables by country.

		Country						
Age Group		AT	DE	ES	IT	PL	Total	Chi-Square
18–35 years	*n* (%)	747 (69.9%)	4969 (73.9%)	3072 (69.4%)	895 (74.6%)	1062 (66.4%)	10,745 (71.6%)	Χ² (8, *n* = 15,015) = 63.49 *p* < 0.001
36–55 years	*n* (%)	288 (26.9%)	1610 (24.0%)	1239 (28.0%)	267 (22.3%)	485 (30.3%)	3889 (25.9%)	
>55	*n* (%)	34 (3.2%)	143 (2.1%)	114 (2.6%)	37 (3.1%)	53 (3.3%)	381 (2.5%)	
Total	*n* (%)	1069 (100.0%)	6722 (100.0%)	4425 (100.0%)	1199 (100.0%)	1600 (100.0%)	15,015 (100.0%)	
Gender								Chi-Square
Female	*n* (%)	750 (70.2%)	4628 (68.8%)	2991 (67.6%)	765 (63.8%)	1143 (71.4%)	10,277 (68.4%)	Χ² (4, *n* = 15,015) = 22.04 *p* < 0.001
Male	*n* (%)	319 (29.8%)	2094 (31.2%)	1434 (32.4%)	434 (36.2%)	457 (28.6%)	4738 (31.6%)	
Total	*n* (%)	1069 (100.0%)	6722 (100.0%)	4425 (100.0%)	1199 (100.0%)	1600 (100.0%)	15,015 (100.0%)	

**Table 2:** Patients’ self-motivation by country.

Country								
Self-Motivation		AT	DE	ES	IT	PL	Total	Chi-Square
Crowding	*n* (%)	386 (36.1%)	2231(33.2%)	2005 (45.3%)	505 (42.1%)	600 (37.5%)	5727 (38.1%)	Χ² (16, *n* = 15,015) = 1370.83 *p* < 0.001
Other, dental health/functional	*n* (%)	244 (22.8%)	1289 (19.2%)	1459 (33.0%)	355 (29.6%)	324 (20.3%)	3671 (24.5%)	
Protruding teeth	*n* (%)	247 (23.1%)	1766 (26.3%)	447 (10.1%)	194 (16.2%)	272 (17.0%)	2926 (19.5%)	
Spacing	*n* (%)	125 (11.7%)	1000 (14.9%)	463 (10.5%)	132 (11.0%)	133 (8.3%)	1853 (12.3%)	
N/a	*n* (%)	67 (6.3%)	436 (6.5%)	51 (1.1%)	13 (1.1%)	271 (16.9%)	838 (5.6%)	
Total	*n* (%)	1069 (100.0%)	6722 (100.0%)	4425 (100.0%)	1199 (100.0%)	1600 (100.0%)	15,015 (100.0%)	

**Table 3:** Oral dental health issues by country.

		Country						
CMD		AT	DE	ES	IT	PL	Total	Chi-Square
No	*n* (%)	1069 (100.00 %)	6697 (99.63%)	4413 (99.73%)	1196 (99.75%)	1596 (99.75%)	14,971 (99.71%)	Χ² (4, *n* = 15,015) = 4.822 *p* = 0.306
Yes	*n* (%)	0 (0.00%)	25 (0.37%)	12 (0.27%)	3 (0.25%)	4 (0.25%)	44 (0.29%)	
Periodontitis							Total	
No	*n* (%)	1063 (99.44%)	6680 (99.38%)	4327 (97.79%)	1183 (98.67%)	1588 (99.25%)	14,841 (98.84%)	Χ² (4, *n* = 15,015) = 65.795 *p* < 0.001
Yes	*n* (%)	6 (0.56%)	42 (0.62%)	98 (2.21%)	16 (1.33%)	12 (0.75%)	174 (1.16%)	
Carious lesions							Total	
No	*n* (%)	1067 (99.81%)	6679 (99.36%)	4291 (96.97%)	1193 (99.50%)	1585 (99.06%)	14,815 (98.67%)	Χ² (4, *n* = 15,015) = 140.255 *p* < 0.001
Yes	*n* (%)	2 (0.19%)	43 (0.64%)	134 (3.03%)	6 (0.50%)	15 (0.94%)	200 (1.33%)	
Total	*n* (%)	1069 (100.00%)	6722 (100.00%)	4425 (100.00%)	1199 (100.00%)	1600 (100.00%)	15,015 (100.00%)	

**Table 4:** Missing teeth by country.

		Country						
# Teeth Missing		AT	DE	ES	IT	PL	Total	Chi-Square
1–2 teeth missing	*n* (%)	6 (0.56%)	133 (1.98%)	181 (4.10%)	8 (0.67%)	30 (1.88%)	358 (2.38%)	Χ² (12, *n* = 15,015) = 116.281 *p* < 0.001
3–5 teeth missing	*n* (%)	2 (0.19%)	59 (0.88%)	48 (1.09%)	1 (0.08%)	10 (0.63%)	120 (0.80%)	
>5 teeth missing	*n* (%)	0 (0.00%)	6 (0.09%)	1 (0.02%)	0 (0.00%)	2 (0.13%)	9 (0.06%)	
No teeth missing	*n* (%)	1061 (99.25%)	6524 (97.05%)	4195 (94.80%)	1190 (99.25%)	1558 (97.38%)	14,528 (96.76%)	
Total	*n* (%)	1069 (100.00%)	6722 (100.00%)	4425 (100.00%)	1199 (100.00%)	1600 (100.00%)	15,015 (100.00%)	

## Data Availability

The data are available on reasonable request due to privacy restrictions.

## Appendix A

**Table A1:** Patient self-motivation by gender.

		Gender			
Self-Motivation		Female	Male	Total	Chi-Square
Crowding	*n* (%)	4012 (39.04%)	1715 (36.20%)	5727 (38.14%)	Χ² (4, *n* = 15,015) = 103.29 *p* < 0.001
Other, dental health/functional	*n* (%)	2494 (24.27%)	1177 (24.84%)	3671 (24.45%)	
Protruding teeth	*n* (%)	2096 (20.40%)	830 (17.52%)	2926 (19.49%)	
Spacing	*n* (%)	1088 (10.59%)	765 (16.15%)	1853 (12.34%)	
N/a	*n* (%)	587 (5.71%)	251 (5.30%)	838 (5.58%)	
Total	*n* (%)	10,277 (100.00%)	4738 (100.00%)	15,015 (100.00%)	

**Table A2:** Insurance types by country.

		Country						
Insurance Type		AT	DE	ES	IT	PL	Total	Chi-Square
Private	*n* (%)	103 (9.64%)	696 (10.35%)	1331 (30.08%)	196 (16.35%)	312 (19.50%)	2638 (17.57%)	Χ² (16, *n* = 15,015) = 17.78 *p* < 0.001
Statutory	*n* (%)	899 (84.10%)	5590 (83.16%)	3043 (68.77%)	990 (82.57%)	1017 (63.56%)	11,539 (76.85%)	
N/a	*n* (%)	67 (6.27%)	436 (6.49%)	51 (1.15%)	13 (1.08%)	271 (16.94%)	838 (5.58%)	
Total	*n* (%)	1069 (100.00%)	6722 (100.00%)	4425 (100.00%)	1199 (100.00%)	1600 (100.00%)	15,015 (100.00%)	

**Table A3:** Missing teeth by age group and gender.

		Age Group			
# Teeth Missing		18–35 Years	36–55 Years	>55	Chi-Square
1–2 teeth missing	*n* (%)	197 (1.83%)	140 (3.60%)	21 (5.51%)	Χ² (6, *n* = 15,015) = 117.897 *p* < 0.001
3–5 teeth missing	*n* (%)	58 (0.54%)	51 (1.31%)	11 (2.89%)	
>5 teeth missing	*n* (%)	1 (0.01%)	8 (0.21%)	0 (0.00%)	
No teeth missing	*n* (%)	10,489 (97.62%)	3690 (94.88%)	349 (91.60%)	
Total	*n* (%)	10,745 (100.00%)	3889 (100.00%)	381 (100.00%)	
		**Gender**			
**# Teeth Missing**		**Female**	**Male**		**Chi-Square**
1–2 teeth missing	*n* (%)	253 (2.46%)	105 (2.22%)		Χ² (3, *n* = 15,015) = 1.812 *p* = 0.612
3–5 teeth missing	*n* (%)	86 (0.84%)	34 (0.72%)		
>5 teeth missing	*n* (%)	7 (0.07%)	2 (0.04%)		
No teeth missing	*n* (%)	9931 (96.63%)	4597 (97.02%)		
Total	*n* (%)	10,277 (100.00%)	4738 (100.00%)		
